# Morbid Sympathies of the Teeth

**Published:** 1851-07

**Authors:** John A. Johns

**Affiliations:** Pleasant Grove, Va.


### ARTICLE VI.

### *Morbid Sympathies of the Teeth.* By JOHN A. JOHNS, M. D., D. D. S. *Pleasant Grove, Va.*

IF we cast our eye over the human body, we see that the latter is made up of a series of separate and distinct organs or parts, each designed to execute a separate and distinct function, for which its structure and relations are admirably adapted. These multiple organs, differing in every possible way from each other, make up one complex and harmonious whole, (of which, each organ forms an indispensable part,) and is endowed with what Bichat is pleased to denominate "an aggregate of those functions by which death is resisted"—i. e. life. Between all these organs or parts, there exists an intimate relation and mutual dependence, each one taking cognizance, as it were, of the condition of all the rest; and hence no organ can be diseased long without involving others. This sympathy between one part and another, situated at a distance, is due to the peculiar endowment of the nervous system, by virtue of which, it transmits sensations and impressions, and if all the organs of the body are not dependent on it for the discharge of their respective functions, they are greatly influenced by it, as the functions of circulation and respiration.

These sympathies may be propagated or extended from one organ to another in several different ways, the principle of which are, reflex action, continuous sympathy, contiguous sympathy, or in a way which, in the present state of our knowledge, is not very explicable. And in some of the lower animals, parts sympathise where there is obviously no nervous centre and probably no nerves. (Dunglison.) Some important functions are performed through reflex action, as the function of deglutition, the contractions of the iris in responding to the impressions of light made on the retina. As examples of continuous sympathy, may be mentioned the effects of diseased teeth and gums on the mucous lining of the stomach and intestines. As examples of contiguous sympathy, may be mentioned the influence of substances in the duodenum, stimulating the liver to action, and cathartics acting on the rectum exciting the uterine system.

Sympathies may also be remote, as between the internal mucous membrane and skin (this is termed remote sympathy, though the membranes are actually continuous) which is evinced by the profuse sweats that follow the introduction of draughts of water into the stomach. There is also an evident sympathy between the kidneys and skin, also between the lungs and skin.

Organs also sympathize through similarity of functions, hence one eye cannot be seriously diseased without affecting the other, though this is not the only way the eyes are sympathetically connected. This remark is also applicable to the kidneys, and to some extent to the teeth.

Nor are these sympathies less active during a morbid, than during a healthy, condition; a knowledge of which must form part of the basis of all rational practice, and without the dental surgeon and practitioner would have to content themselves with a practice strictly empyrical. And possibly a knowledge of morbid sympathies can never do more good to fallen humanity, or bring more credit to the practitioner than when applied to the teeth, as these organs have quite an extensive range of sympathies, influencing the general health sometimes to an extent little suspected. And on the other hand, they readily take on morbid action through sympathy with the general system, in a manner we will explain presently. This is particularly the case during their formation, as during this period the system is more irritable and the symptoms more active than in after life. At this period, disease may be stamped on them which can never be eradicated, owing to their peculiar and inherent laws of organization, differing in this respect from other tissues, which may wholly or partially recover from disease impressed on them during their formation, or during utero-gestation. Diseases may be transmitted hereditarily in the teeth as in other organs.

The constitution of the teeth may vary in different individuals from a predominance or deficiency of either the earthy or animal constituents, owing to an interruption in the nutritive function during their formation. When there is a predominance of the earthy salts, they rarely decay, and are of a dull white color, short and thick. But when the earthy material is deficient, the animal predominating, they are readily acted on chemically, and hence may be influenced by any disease that produces a change in, or vitiates the secretions of the mouth. Teeth of this description are very liable to atrophy. It is probable that the denuding process depends in a great part on imperfection in the formation of the teeth. The permanent teeth may also be morbidly impressed during their early development by disease of the deciduous teeth being extended to the pulps or investing membrane of the former. There are other affections of the teeth, such as abnormal relation and position, a third set, &c. which must be classed rather as freaks of nature, than referred to any determined or known cause, or condition of the system.

This imperfection in the constitution and organization of the teeth is readily accounted for by remembering that they are secreted from the blood, and that the condition of this fluid is always influenced by any disease involving the system at large, deranging the function of the chylopoetic viscera. This is very liable to occur in hereditary predisposition to strumous or phthisical diseases. The occurrence of acute diseases, as measles, small pox, pertussis, fevers, &c. may so derange the function of nutrition, as to impress disease on the particular teeth that are undergoing ossification at the time, while the other teeth may be healthfully developed. In this way we account for the disposition of the teeth to decay in pairs.

Although the teeth during life participate in the healthy or diseased action of the general system, yet this does not occur on account of any change that takes place in the physical organization or structure of the tooth, but by the change produced in the fluids of the mouth, causing them to act on the teeth chemically. This is evident from the fact that when a tooth is once formed it remains permanent, not being subject to the laws of disintegration and renewal as other structures are.

There are many other diseases of the teeth and gums associated with constitutional disease, such as the effects of mercury on the gums and alveolar processes, protrusion of the teeth by deposition of ossific matter in their sockets, mechanical injuries of the teeth, &c. but which do not come within the scope of this article.

Having considered the extensive and intimate sympathetic relation of the various organs of the body, and the morbid relation of the teeth and system at large, as regards the influence of the latter, in a diseased condition, on the former, there next remains to be considered the influence of the former in a diseased state on the latter.

Some of the diseases of the teeth affect the general health very little, if at all, as the denuding process, atrophy, &c., but unfortunately this is far from being true of all, as diseases of these organs are readily propagated or extended to other parts, involving particularly the nervous and vascular system. Nor is this at all strange when we bear in mind the importance of these organs in preparing the food for digestion, as to them is trusted the functions of tearing and comminuting the food that it may be mixed with saliva of the mouth, (which is an important step in the process of digestion,) and more readily dissolved by the juices of the stomach. And disease of these organs readily affects the salivary glands, deranging their function and rendering their secretion unfit for the use for which it is designed. This fluid may contain an abnormal quantity of earthy salts, which being deposited on the teeth as salivary calculi, produces disease, irritation and inflammation of the gum, and mucous membrane which may result in loss of the teeth. And these diseases of the teeth and gums, connected with incapacity on the part of the teeth to prepare the food for its reception into the stomach, may, and often does give rise to disease of the latter, and in fact to the whole digestive apparatus; hence dyspepsia, emaciation, depressed spirits, melancholy, &c. And thus a condition of the system which may predispose it to any of the diseases common to fallen humanity. And all this train of morbid phenomena may be traceable to a single carious or necrosed tooth. The nervous system of females sometimes becomes so irritable through sympathy with the diseases of the teeth, that the slightest excitement cannot be borne without the occurrence of serious nervous symptoms.

Of the diseases produced by or connected with the teeth, one not to be overlooked, is neuralgia. This affection often takes its origin in a carious tooth and is a source of an immense deal of pain and annoyance to the patient. The indication clearly is to remove the offending member. The manner in which a diseased tooth may produce neuralgia of the face becomes quite plain, when we remember that the same nerve gives branches to both, the affection being reflected along the branch supplying the former, and is reflected along the branch supplying the latter, giving rise to pain in any part supplied by it. Many such instances of disease are found in the system—thus the phrenic nerve gives some branches to the shoulder, and also communicates with the nerves supplying the cœliac aris, and in this way is explained the fact that in disease of the liver we often have pain at the top of the shoulder. The pneumogastric nerve, from its relations, may have something to do in the production of this phenomenon.

During dentition the liability to disease is very much increased, the disturbance from this cause frequently producing engorgement of the brain, depraved nutrition and sometimes convulsions. And where there is an hereditary predisposition to phthisis or struma, the constitutional disturbance, produced by dentition, acts as a powerful exciting cause to their development. The ravages of any disease, cæteris paribus, are much more dangerous, occurring during dentition, than at any other period of life, except probably, in extreme old age.

The constitutional and local disturbance produced by disease of the teeth, are beautifully summed up by a modern writer as follows: "Affections of the teeth are not connected only with the inconvenience, co mfrt and good looks of a patient—but with his health and very existence. The cause sometimes remote, sometimes tolerably direct—of many affections, implicating the general frame, as well as important parts of it, proceed entirely from the contents of the alveoli. Bad teeth are frequently the cause—the sole cause of violent and continued headache, of glandular swellings in the neck, terminating in or combined with abscess; of inflammation and enlargement of the tonsils, either chronic or acute; of ulceration of the tongue or lips, often assuming a malignant action from continued irritation; of painful swellings in the face, tic douloureux, pains in the tongue, jaws, &c.; of abscess and sinus of the cheek; of enlargement and change of structure in the gum, which may lead to dangerous tumors of the bone; of disorder of the stomach, from affection of the nerves, or from imperfect mastication; and of continued constitutional irritation, which may give rise to serious constitutional disease."

